# A systematic review of post-traumatic growth in ambulance personnel: facilitators and prevalence rates

**DOI:** 10.29045/14784726.2024.6.9.1.34

**Published:** 2024-06-01

**Authors:** Molly Abdo, Annette Schlösser

**Affiliations:** Humber Teaching NHS Foundation Trust ORCID iD: https://orcid.org/0009-0004-7662-9363; University of Hull ORCID iD: https://orcid.org/0000-0001-5478-0856

**Keywords:** ambulance personnel, paramedic, PTG

## Abstract

**Introduction:**

Ambulance personnel are exposed to traumatic and stressful situations, which can increase the risk of mental health conditions, such as post-traumatic stress disorder (PTSD). High rates of PTSD have been found in ambulance personnel (Petrie et al., 2018), but no review is available to examine post-traumatic growth (PTG - positive psychological change following a trauma) in this population. This literature review provides an overview of the prevalence rates and facilitators that may contribute to PTG in ambulance personnel.

**Methods:**

A systematic search was conducted on EBSCOhost in January 2024 across the following six databases: Academic Search Ultimate, PsycINFO, PsycARTICLES, MEDLINE, ERIC and Cumulative Index to Nursing and Allied Health Literature (CINAHL) Ultimate.

**Results:**

Eleven papers were identified for this review. Pooled prevalence of PTG was moderate (52%), and facilitators for PTG were grouped into five categories: coping style/strategies, resilience, personality traits, gender and incident characteristics.

**Conclusions:**

Numerous facilitators contributed to the development of PTG, although these did not arise in all papers. The quality of research ranged from satisfactory to excellent. Evidence suggested that adaptive coping style, high levels of resilience, the absence of a personality trait (neuroticism) and being female may facilitate PTG. Further research is needed to support the reliability of findings.

## Introduction

Frontline ambulance personnel are exposed to stressful and traumatic events. These include medical emergencies, road traffic collisions and suicide (Alexander & Klein, 2001). This increases the risk of poor mental health, such as depression, anxiety and PTSD (Lawn et al., 2020). However, a concept known as *post-traumatic growth* (PTG) has also been explored in the literature since being coined in the mid-1990s (Tedeschi & Calhoun, 1995). Tedeschi and Calhoun defined PTG as ‘positive psychological changes experienced as a result of the struggle with traumatic or highly challenging life circumstances’ (Tedeschi et al., 2018, p. 3). These domains relate to longer-term changes over a period of days to years after the trauma and reflect situations ‘where people develop new ways of thinking, feeling, and behaving’ as a result of challenges to their assumptive world and core beliefs (Tedeschi et al., 2018, p. 5). These changes have been described as a ‘personality transformation’ (Tedeschi et al., 2018, p. 11), characterised by periods of growth due to positive and personal development in five domains of life: ‘relating to others, new possibilities, personal strength, spiritual change, and appreciation of life’ (Tedeschi & Calhoun, 1996, p. 5). PTG is therefore perceived as both a process and an outcome, and is distinguished from the concept of resilience (‘bouncing back’ from trauma (American Psychological Association, 2022, para. 4)), as PTG is a measurable and marked change from an individual’s previous baseline of functioning.

Tedeschi et al. (2018) posited a model of PTG wherein the person’s pre-trauma demographics – such as age (working age to older adult), gender (female) and religiosity – and individual differences – such as personality traits, including hope, extraversion and openness, and well-adjusted mental health – are associated with higher levels of PTG and are expected to impact how the person experiences the traumatic event(s). If one’s core beliefs are challenged, PTG can be facilitated through intrusive rumination and coping, which can reduce emotional distress by engaging in cognitive processing. Therefore, PTG can be influenced by proximal (immediate social systems, such as family and neighbourhood) and distal (communities, such as workplace and worship places) cultural systems (Bronfenbrenner, 1979). Most ambulance personnel in the United Kingdom (UK) work for the publicly funded National Health Service (NHS) within ambulance service trusts, providing pre-hospital treatment and/or advice to patients in the community and social care provision (NHS Providers, 2019). They face significant challenges in the workplace, such as physical and verbal abuse (Murray et al., 2020), witnessing life-threatening injuries, death and suicide (Alexander & Klein, 2001) and working long shifts (Kirby et al., 2016). These challenges could be experienced as traumatic, and are the likely cause for ambulance personnel in the UK having the highest rates of stress-related sickness absence compared to other healthcare professions (NHS Digital, 2021). This has implications for clinical psychologists working in the NHS, who could shift their attention from a problem-focused approach and mental ill-health, to becoming an ‘expert companion’, by acknowledging and understanding the trauma reaction, while guiding ambulance workers’ transformation to PTG using a strength-based approach (Tedeschi et al., 2018). Furthermore, PTG was recently reported in a large-scale systematic review by Henson et al. (2021), who investigated PTG in diverse populations within different contexts. Coping style, resilience, personality traits and gender contributed to PTG in samples including firefighters, HIV-positive individuals, cancer survivors, natural disaster survivors, sexual assault victims, veterans, refugees, students, bereaved adults, medical staff, homeless women, police officers and parents of children with severe illness. However, no systematic review has explored PTG in ambulance personnel.

### Aim and research questions

The aim of this literature review is to provide a comprehensive review and synthesis of research studies exploring PTG in ambulance personnel, which may have implications for ambulance services and clinical psychology. A systematic review was chosen due to a gap in the evidence base in terms of necessary identification, appraisal and synthesis of all relevant studies using a rigorous approach. The operational definition of ambulance personnel in this review includes samples of emergency medical technicians (EMTs), paramedics and other staff manning ambulances, the latter being typical in collectivist societies. Student paramedics were excluded from the review, as the authors wanted to capture research that included qualified and experienced personnel for the purpose of homogeneity.

The following questions guided this systematic review:

1. What is the reported prevalence (low, moderate or high) of PTG in ambulance personnel?
2. Which facilitators contribute to PTG in ambulance personnel?

## Methods

The Preferred Reporting Items for Systematic Reviews and Meta-analyses (PRISMA) (Moher et al., 2009) and PICO tool (Schardt et al., 2007) were utilised. The population (P) was ambulance personnel, the intervention (I) was the facilitators of PTG and there was no comparator (C) used to achieve the outcome (O), which was a review of the combined prevalence and facilitators of PTG in ambulance personnel.

### Search strategy

The search was carried out in January 2024, and papers from January 2000 to January 2024 were reviewed. This captured papers published at the beginning of the 21st century, coinciding with the positive psychology movement (Csikszentmihalyi & Seligman, 2000) and usage of the term PTG (Tedeschi & Calhoun, 1995). However, the temporal range of published papers varied from 2003 to 2021. The search strategy included the following keywords and terms divided into two categories, concept and role, respectively:

( (“post traumatic growth” or “post-traumatic growth” or PTG or “posttraumatic growth” or “stress-related growth” or “stress related growth” or “adversarial growth” or “positive psychological chang*”) or ((“improv*” or “positiv*”) N3 (“trauma*”)) ) AND (“paramedic*” or “emergency medical service*” or “EMS” or “prehospital*” or “pre-hospital*” or “ambulance*” or “emergency medical technician*” or “EMT” )

### Sources of information

The search strategy yielded 1357 articles from six databases: Academic Search Ultimate, CINAHL Ultimate, MEDLINE, APA PsycARTICLES, APA PsycINFO and ERIC. The limiters ‘journal article’ and ‘English language’ were applied, and duplicates were removed, which resulted in 992 journals for screening.

### Screening and selection

**Figure 1:**
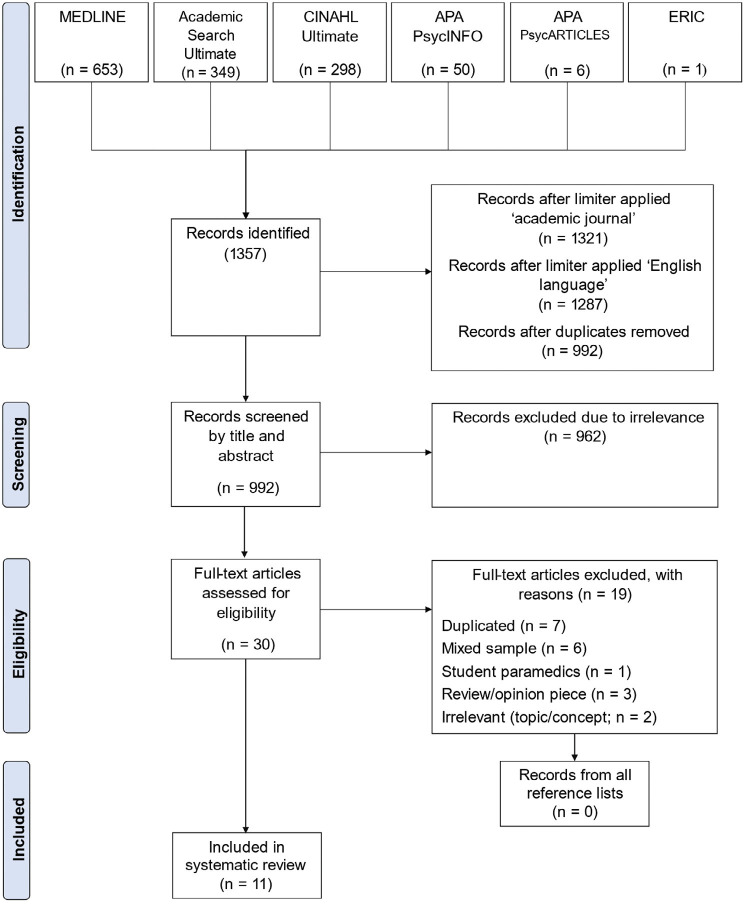
Figure 1. Adapted from ‘PRISMA 2009 Flow Diagram’ (Moher et al., 2009).

After title and abstract screening, 30 studies were retained. Further screening revealed seven duplicates, six papers with a combined sample (data pooled from first responders including ambulance, fire and police)), one with a student sample (Miller et al., 2020), three review/opinion pieces and two that were irrelevant. These papers did not meet the inclusion criteria. See Table 1 for inclusion and exclusion criteria. The reference lists of the final pool were screened, and no new relevant publications were discovered. This left 11 papers that met full eligibility criteria. The reviewer contacted the authors of the excluded papers (with a combined sample) to request whether the data could be extrapolated for ambulance personnel alone. However, no further correspondence was received. See Figure 1 for the PRISMA diagram.

**Table 1.** Inclusion/exclusion criteria.

Inclusion	Exclusion
Sample specific to paramedic/emergency medical service (EMS) populations	Sample not specific to paramedic/EMS-based populations (e.g. fire or police)
Mixed sample that includes EMS workers who have physical contact with the patient pre-hospital	Mixed sample that includes emergency service (e.g. ambulance AND fire AND/OR police) workers who have contact with the patient pre-hospital or in a hospital setting
Studies including employed and/or volunteer paramedics/EMS role	Studies including an undergraduate student population
Outcomes related to the prevalence and/or facilitators of PTG	Outcomes related to intervention or only PTSD or other mental health outcome
Published in the English language	Published in a language other than English
Published January 2000-January 2024	Published prior to 2000
Studies with quantitative or qualitative data	Opinion pieces or dissertation previews

### Quality review

The methodological quality of each paper was assessed using the Mixed Methods Appraisal Tool (MMAT) (Hong et al., 2018). The MMAT contains two screening questions, both of which a paper must pass, followed by five further questions. The papers in this review were either quantitative (descriptive) or qualitative, and were assigned a ‘yes’, ‘no’ or ‘cannot tell’ rating based on the following questions:

Quantitative (descriptive):

1. Is the sampling strategy relevant to address the research question?
2. Is the sample representative of the target population?
3. Are the measurements appropriate?
4. Is the risk of non-response bias low?
5. Is the statistical analysis appropriate to answer the research question?

Qualitative:

1. Is the qualitative approach appropriate to answer the research question?
2. Are the qualitative data collection methods adequate to address the research question?
3. Are the findings adequately derived from the data?
4. Is the interpretation of results sufficiently substantiated by data?
5. Is there coherence between qualitative data sources, collection, analysis and interpretation?

Each paper is graded out of 5, with the maximum score indicating high quality. Methodological quality in the sample ranged from 3 to 5 (satisfactory to excellent), with a mode of 5. See Table 2 for quality appraisal at item level. To assess the scoring inter-rater reliability, all 11 papers were first marked independently by the lead author (MA) and then marked blindly by a peer (psychology research master student) to check for consistency of scoring. There was 87% inter-rater reliability, and difference in scoring was resolved following discussion.

**Table 2.** Quality appraisal scores.

Author	Quantitative (descriptive)					Qualitative					Overall quality rating
	4.1	4.2	4.3	4.4	4.5	1.1	1.2	1.3	1.4	1.5	
Austin et al. (2018)	No	Yes	Yes	No	Yes						3
Avȿarolu (2019)	Yes	No	Yes	Yes	Yes						4
Jurišová (2016)	Yes	Yes	Yes	Yes	Yes						5
Kang et al. (2018)	Yes	Yes	Yes	Yes	Yes						5
Kirby et al. (2011)	Yes	Yes	Yes	Yes	Yes						5
Ogińska-Bulik and Kobylarczyk (2015)	No	No	Yes	Yes	Yes						3
Ragger et al. (2019)	Yes	Yes	Yes	Yes	Yes						5
Shakespeare-Finch et al. (2003)	Yes	Yes	Yes	Yes	Yes						5
Shakespeare-Finch et al. (2005)	Yes	Yes	Yes	Yes	Yes						5
Surgenor et al. (2020)	No	Yes	Yes	No	Yes						3
Wines (2016)						Yes	Yes	Yes	Yes	Yes	5

## Results

### Characteristics of included studies

Ten of the papers utilised a quantitative methodology and one study employed semi-structured interviews to collect qualitative data. The cumulative sample included 2324 ambulance workers from 11 studies, which represented eight countries across four continents (USA, Australia, Europe and Asia). The age of participants ranged from 18 to 75 and duration of service ranged from six weeks to fifty-two years. The range was reported rather than the mean due to some studies not reporting age or duration. See Table 3 for included study characteristics and overall quality scores.

**Table 3.** Characteristics of the included studies.

No.	Author(s) and date	Study location	Sample size (N)	Target population	Gender	Age	Duration of service	Inclusion criteria	Methodology	Measures	Prevalence of PTG	Overview of findings	Bias	Quality rating (MMAT)
1	Austin et al. (2018)	USA	54	Paramedics (61%) and EMTs* (39%)	NR* –’majority male’	NR	5 ≥ 10 years (M =10.61; SD = 9.15)	NR	Quantitative	Post-traumatic growth inventory (PTGI); Brief Resilience Scale (BRS); Changes in Outlook Questionnaire-Short (COIQ-S)	M = 47.87; SD = 24.12 [0, 100]	EMTs had higher PTG than paramedics. Positive change in outlook was correlated (+) with PTG. Participants interested in coping training had higher resiliency and growth scores.	Selection bias from convenience sampling and some self-report questionnaires being distributed by supervisors.	**3**
2	Avşarolu (2019)	Kyrgyzstan	400	Emergency health employees	Male and female	NR	NR	NR	Quantitative	PTGI; The Hopelessness Scale; Locus of Control Scale, Problem Solving Inventory; Multidimensional Scale of Perceived Social Support; Peritraumatic Dissociative Experiences Questionnaire-R	Average = 37.57/105, SD = 25.45, [0,105]	Incident effect significantly predicted PTG positively for both genders.	None identified or disclosed.	**4**
3	Jurišová (2016)	Slovakia	62	Paramedics	Male and female (30 and 32)	21–53 (M = 35.91; SD = 8.97)	1–34 years (M = 7.54; SD = 5.79)	NR	Quantitative	PTGI; COPE (Slovak version); General Self-efficacy scale (GSE); Positive and Negative Affect Scale (PANAS)	Average = 55.87, SD = 18.72, [21, 94]	Average levels of PTG were correlated with most coping strategies. Self-efficacy and (+) affectivity moderated the relation between coping and PTG.	None identified or disclosed.	**5**
4	Kang et al. (2018)	China	227	Nurses (103); physicians (71) and other ambulance workers (53; drivers and stretcher bearers)	Male and female (48.5% male)	22–55 years (M = 31.76; SD = 6.52)	≤5 years 128 (56.4%) >5 years 99 (43.6%)	Minimum 3 months working on an ambulance	Quantitative	PTGI; Social Support Rating Scale (SSRS; Chinese version) 10-Item Connor-Davidson Resilience Scale (CD-RISC-10)	M = 68.96; SD = 15.51	Subjective support, objective support and support-seeking behaviours were (+) related to vicarious PTG (VPTG). Resilience was (+) associated with VPTG (r = 0.670, p < 0.01), which mediated the relationship between social support and VPTG.	Possible reporting bias as vicarious PTG was measured by the PTG scale as no instrument was available to measure VPTG in addition to selection bias from convenience sampling.	5
5	Kirby et al. (2011)	Australia	118	Paramedics	Male and female (78 and 40)	18–61 (M = 37; SD = 10.49)	6 weeks – 39 years (M = 10 years; SD = 9.32 years)	Experience of a traumatic event; Minimum of 4 years ‘on-road’ experience	Quantitative	PTGI; R-COPE	Range = 40.56–58.57	Adaptive coping strategies were predictive of higher scores on the PTGI subscales of Spiritual Change and Relating to Others with a positive trend to Personal Strength subscale. PTG was the highest when the victim was known to the paramedic.	Self-report vulnerable to social desirability bias.	5
6	Ogińska-Bulik and Kobylarczyk (2015)	Poland	80	Paramedics	Male	21–67 (M = 35.47; SD = 10.21)	NR	Experience of a traumatic event in the past 5 years	Quantitative	PTGI (Polish version); Inventory mini-cope; Assessment Resiliency Scale	M = 68.52; SD = 17.99	Common strategies were active coping and planning. Significant changes relate to Self Perception and Appreciation of Life subscales. Planning is a mediator between PTG and resiliency. Denial and venting reduce resiliency and suppress PTG.	Research conducted during the performance of duties by a seminar participant who may have been known to respondents. Self-report also vulnerable to social desirability bias.	3
7	Ragger et al. (2019)	Austria	266 (216 voluntary and 50 full time)	Critical care paramedics (68) and EMTs (198)	Male and female (179 and 87)	18–73 (M = 29.94; SD = 11.07)	1–52 years (M = 9.91; SD = 9.04)	NR	Quantitative	PTGI; Sense of Coherence Scale (SOC-29)	M = 43.38; SD = 15.03	Sense of coherence was significantly correlated to PTG. The SOC subscale Meaningfulness correlated (+) with overall PTG and subscales New Possibilities, Relating to Others and Appreciation of Life.	None identified or disclosed.	5
8	Shakespeare-Finch et al. (2003)	Australia	526	Ambulance officers	Male and female (423 and 103)	21–63 years (M = 39.84; SD = 9.41)	1–43 years (M = 11.58; SD = 8.17)	New officers had no previous exposure to work-related trauma	Quantitative	PTGI	M = 49.08 (SD = 21.53, range = 0–100; seasoned officer); M = 42.45 (SD = 26.05, range = 0–84; new recruits)	Ambulance officers with a trauma in their personal lives and at work had higher levels of PTG. Seasoned officers had higher PTG than new officers.	Out-dated personnel records precluded every participant from being sampled (197/1914 questionnaires were unopened and returned to sender). However, overall response rate was typical at 31%.	5
9	Shakespeare-Finch et al. (2005)	Australia	526	Operational ambulance officers	Male and female (423 and 103)	21–63 (M = 39.84; SD = 9.41)	1–43 years (M = 11.58; SD = 8.17)	NR	Quantitative	Neuroticism Extraversion Openness-Five Factor Inventory (NEO-FFI); Coping Responses in Rescue Workers Inventory (CRRWI); PTGI	Mc = 51.19; SDc = 21.36 [0, 100]	Extraversion, openness, agreeableness, conscientiousness and coping levels significantly relate to PTG. The relationship between personality and PTG is mediated by levels of coping.	None identified or disclosed.	5
10	Surgenor et al. (2020)	New Zealand	579	Paramedics (37.90%); intensive care paramedics (29.20%); EMTs (13.80%); clinical hub/ambulance nurses (10.40%) and other <10%. Professional (70.46%) and volunteer (29.54%)	Male and female (51.80% male)	18–75 (M = 42.28; SD = 12.50)	M = 10.90; SD = 8.90	Active first responders	Quantitative	PTGI-SF; Core Beliefs Inventory (CBI)	The majority (66.40%) of participants had some degree of PTG total score >10	Being female and experiencing greater core belief disruption independently contributed to higher levels of PTG.	Data collection went live on the day of a major earthquake followed by unrelated industrial disputes. Data collection was relaunched, however non-response bias was possible as response rate was 27%.	3
11	Wines (2016)	USA	12	Paramedics (7) and EMTs (5)	Male and female (8 and 4)	24–57	7–37 years (M = 18)	Certified EMT or paramedic; employed or volunteered for at least one year: at least 18 years old; responded to at least one suicide call with a loved one present	Qualitative – hermeneutic phenomenology	Semi-structured interview	NA	(1) Risk factors, (2) Protective factors, (3) Lived existential and (4) Meaning. The theme of detachment was common amongst 1 & 2.	Possible bias through social desirability and researchers’ own biases and interpretation of qualitative data. This may have been minimised through analytic note taking and consultation with committee members.	5

NR: not recorded; EMT: emergency medical technician Prevalence of PTG

### Prevalence of PTG

**Figure 2:**
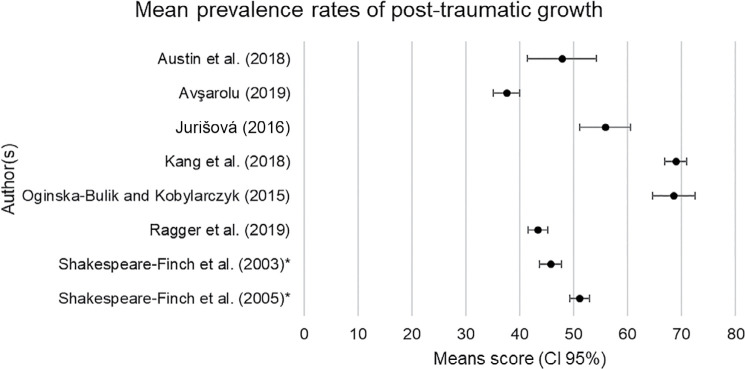
Figure 2. Interval plot showing mean prevalence rates of post-traumatic growth in ambulance personnel. CI = confidence interval (95%); *combined mean.

Mean comparisons are provided for all quantitative studies using the Post-Traumatic Growth Inventory (PTGI). Two studies did not use the PTGI, and one study did not provide sufficient data for a mean comparison. The cumulative mean of PTG scores from eight studies was 52% (*SD* = 10.7, 95% *CI* = 44.6, 59.4). The mean and standard deviation (SD) was calculated using the AVERAGE and STDEV.P formulae in Excel, respectively. The SD demonstrates a large variance in relation to the mean, which could be due to difference in study characteristics. The prevalence of PTG ranged from low to moderate (38%–69%) in ambulance personnel (see Figure 2).

### Synthesis of findings

The results are presented as a narrative synthesis of mixed methods, with facilitators of coping style/strategies, resilience, personality traits, gender and incident characteristics.

### Coping style/strategies

Seven studies investigated the relationship between coping style or strategies and PTG in ambulance personnel. Kirby et al. (2011) investigated whether coping styles (adaptive or maladaptive) predicted negative or positive post-trauma outcomes (PTSD or PTG) in a group of Australian paramedics. Adaptive coping styles were divided into three subtypes – self-help, approach and accommodation – whereas maladaptive coping styles were divided into two subtypes – avoidance and self-punishment. Results identified that higher scores of adaptive coping styles were significantly predictive of lower scores on the impact of event scale-revised (a measure of subjective distress caused by traumatic events) and higher scores on the PTGI subscales of ‘spiritual change’ (*p* < 0.001, *R*^2^ = 0.12) and ‘relating to others’ (*p* < 0.006, *R*^2^ = 0.08), with a positive trend on the subscale of ‘personal strength’ (*p* = 0.03, *R*^2^ = 0.05). Conversely, higher scores of maladaptive coping styles predicted higher rates of avoidance, hyper-arousal and intrusion on the impact of event scale-revised (*p* < 0.001), which are DSM-IV criteria for the diagnosis of PTSD (American Psychiatric Association, 2013).

Shakespeare-Finch et al. (2005) investigated the relationship between PTG, coping and the Five Factor Model of personality (Costa & McCrae, 1999) in Australian ambulance personnel. They found adaptive coping had a moderate positive relationship with PTG on all subscales, and coping was a mediator of the relationship between personality and PTG. The PTGI subscale of ‘relating to others’ showed the strongest change in PTG (*p* < 0.001, *r* = 0.43), followed by ‘spiritual change’ (*p* < 0.001, *r* = 0.37). These correlations corresponded with the Kirby et al. (2011) study, although unlike the latter study, they did not find significant correlations with all the PTGI subscales.

Jurišová (2016) researched whether coping strategies predicted PTG and if this relationship was moderated by variables of self-efficacy and affectivity in a sample of Slovakian ambulance personnel. They found the highest scores on the PTGI subscales of ‘personal strength’ and ‘appreciation of life’, and several coping strategies were implicated in PTG with correlations ranging from 0.26 (low) to 0.46 (low–moderate). There were significant positive relations between active coping, planning, suppression of competing activities, restraint coping, seeking social support, use of social support, religious coping, focus on and venting of emotions, behavioural disengagement, smoking and PTG, whereas significant negative relations were found between mental disengagement and PTG. The coping strategies with the highest means were acceptance (10.8), followed closely by positive reinterpretation and growth (9.19), and active coping (9.0). Interestingly, the maladaptive coping strategy of behavioural disengagement (avoidance) had a positive correlation with PTG, which is in contradiction with the findings from the Kirby et al. (2011) study, which found a negative correlation between avoidance and PTG. This may be due to differences in interpretation, as within the context of emergency medical response, ‘strategies traditionally referred to as maladaptive may be viewed as a positive way of dealing with a highly stressful scene at the time of the event’ (Kirby et al., 2011, p. 31).

Contradictory to Jurišová’s (2016) findings, Ogińska-Bulik and Kobylarczyk’s (2015) Polish study found that venting of emotions was a suppressor of PTG, rather than an adaptive coping strategy, which was implicated in the former study. Denial was also a suppressor of PTG, a maladaptive coping strategy suggested by Jurišová (2016); although consistent, this was a non-significant finding in Ogińska-Bulik and Kobylarczyk’s (2015) paper.

A Chinese study by Kang et al. (2018) focused on a specific coping strategy: social support (subjective, objective and seeking behaviour), which was implicated as a coping strategy that facilitates PTG (*r* = 0.31–0.43, *p* < 0.01). This is consistent with Jurišová’s (2016) finding of a significant (*p* < 0.05) but low correlation with PTG (*r* = 0.29 (seeking social support) to 0.30 (use of emotional social support)). However, a study in Kyrgyzstan by Avşarolu (2019) did not find a significant correlation between social support and PTG amongst emergency health employees.

Lastly, a qualitative study by Wines (2016) explored the experiences of EMS personnel responding to suicides where loved ones of the deceased were present. Two coping mechanisms were categorised as protective factors: detachment (‘after so long you get used to it’ (p. 100)), and dehumanisation (‘you really distance yourself from the person’ (p. 101)). Task focus was also reported, which could be interpreted as task-orientated coping to divert from emotional stressors when considering the findings of the quantitative papers. A risk factor, intrusive rumination, was also reported (‘I can always picture what they looked like or where we found them . . .’ (p. 99)). This may be interpreted as maladaptive or adaptive coping following trauma. To support the latter hypothesis, Taku et al. (2009) found deliberate or intrusive rumination following a recent trauma was positively associated with PTG, as this may trigger cognitive processing.

Most studies investigating coping style scored a 5 (excellent) on the MMAT, with the exception of Avşarolu (2019) study (4: good) and Ogińska-Bulik and Kobylarczyk’s (2015) study (3: satisfactory). Overall, studies used validated questionnaires and appropriate samples to measure coping.

### Resilience

Three studies investigated the facilitators and relationship between resilience and PTG in ambulance personnel. Kang et al. (2018) found resilience was positively associated with vicarious post-traumatic growth (VPTG) (*r* = 0.67, *p* < 0.01) and was a significant mediator (*r* = 0.58, *p* < 0.001) of the relationship between social support and VPTG (*r* = 0.51, *p* < 0.001), with a small effect size of 0.30. Conversely, Ogińska-Bulik and Kobylarczyk (2015) reported that high levels of resiliency facilitated the use of planning as a coping strategy, which promoted PTG on the ‘appreciation of life’ subscale (*p* < 0.001).

In contrast, Austin et al. (2018) found a small and negative relationship between resilience and PTG (*r* = −0.11). However, high levels of resilience (*p* = 0.001) were present in ambulance personnel who expressed an interest in receiving coping training (support groups or counselling), and this could facilitate or mediate PTG as reported in the wider literature.

Studies that investigated resilience varied in methodological quality, with Kang et al. (2018) scoring a 5 and the two latter studies scoring a 3. This was due to an unrepresentative or unequal sample size, reducing power.

### Gender

Three studies statistically analysed gender. Surgenor et al. (2020) found that female paramedic first responders showed increased levels of PTG (*t* = 4.70, *p* < 0.01). Correspondingly, Shakespeare-Finch et al. (2005) found that female emergency ambulance personnel scored higher on the PTGI (*M* = 54.64; *SD* = 21.28; range = 0–100) than male officers (*M* = 47.74; *SD* = 21.45; range = 0–100; *t* (502) = −2.86, *p* < 0.01).

In Avşarolu’s (2019) study, incident effect on life was the only positive correlation with PTG for both male and female emergency health workers. Although this finding was reported irrespective of gender, the correlation was only highly statistically significant for males (*t* = 4.266, *p* < 0.001 and *t* = 2.655, *p* < 0.01, respectively).

Studies varied in methodological quality from 3 to 5 (Avşarolu (2019), Surgenor et al. (2020) and Shakespeare-Finch et al. (2005), respectively). Two papers utilised an equal sample of both genders. Lower ratings were due to vague or unreported participant demographics apart from sex and mixed samples of volunteers and employees. No other studies found significant correlations between gender and PTG, although most studies included male samples or had a disproportionate sample of males compared to females, limiting the transferability of findings.

### Personality traits

Two studies examined personality. Shakespeare-Finch et al. (2005) highlighted that dimensions of extraversion, openness and conscientiousness were significantly related to all of the PTGI factors, and the dimension of neuroticism did not correlate in either direction with total PTGI score, but did elicit a very weak significant and negative relationship with changes in personal strength. This is consistent with wider literature (Tedeschi & Calhoun, 1996). Sense of coherence (SOC) has been defined in the literature as a personality trait, and Ragger et al. (2019) revealed higher levels of PTG were associated with higher levels of SOC (*r* = 0.27, *p* < 0.01). In particular, the SOC subscale of ‘meaningfulness’ was positively associated with the PTG total score (*r* = 0.27, *p* < 0.01) and with the PTG subscales of ‘new possibilities’ (*r* = 0.27, *p* < 0.01), ‘relating to others’ (*r* = 0.31, *p* < 0.01) and ‘appreciation of life’ (*r* = 0.14, *p* < 0.05). Both studies scored a 5 on methodological quality, and utilised questionnaires (SOC-29 and Five Factor Model) with excellent reliability (0.82–0.95 and 0.77–0.94, respectively) to measure personality (Antonovsky, 1993; Costa & McCrae, 1992).

### Incident characteristics

None of the studies revealed significant correlations between length of service and PTG. However, Ragger et al. (2019) highlighted an increase in PTG when duration of emergency incidents decreased (*r* = −0.16, *p* < 0.01). As previously mentioned, Avşarolu (2019) reported a significant correlation between incident effect and PTG for both genders. Moreover, Kirby et al. (2011) reported that incident type influenced the rate of PTG and use of maladaptive coping, whereas the study by Shakespeare-Finch et al. (2003) demonstrated higher PTG in paramedics who had experienced a personal trauma in addition to a work-related trauma or when the victim was known to the paramedic (*M* = 58.57; *SD* = 24.38), compared to other types of incidents (*F* (4, 113) = 3.77, *p* < 0.01). Other papers in the review did not comment on or capture the demographic variables of employment, incident duration, effect or type. Studies except for Avşarolu (2019) scored a 5 on methodological quality, demonstrating excellent reliability and validity of findings.

## Discussion

### Overview of research findings

The aim of this literature review was to investigate what facilitates PTG in ambulance personnel and to determine a prevalence rate. The synthesis of papers revealed moderate PTG (52%). Facilitators were split into five categories: coping style/strategies, resilience, personality traits, gender and incident characteristics, whereby all but the latter are implicated in a systematic review by Henson et al. (2021).

### Coping style/strategies

Overall, adaptive coping styles were associated with higher levels of PTG than maladaptive coping styles. However, different coping strategies were indicated across papers and there was some overlap in the classification of strategies. For instance, venting of emotions was seen as both a facilitator and suppressor of PTG. The health theory of coping (Stallman, 2020) suggests that western society has conceptualised coping as mutually exclusive and adaptive. However, smoking may be seen as a maladaptive strategy that could facilitate venting of emotions with other smokers, which has an adaptive function and suggests that coping can be perceived as non-mutually exclusive. This demonstrates that coping is not clear cut and questions the reliability of findings produced from quantitative measures.

### Resilience

Resilience was either positively or negatively associated with PTG. PTG was a significant mediator of the relationship between social support and VPTG. High levels of resilience were found in ambulance personnel who expressed an interest in receiving coping training. Resilience theory (Fergus & Zimmerman, 2005) posits that the ability to bounce back from stressors is dependent on availability of resources and assets in multiple contexts and throughout the lifespan. Social support is an important resource that allows an individual to learn ways of coping with stress from resilient role models (Bandura, 1977).

### Personality traits

Three factors of the Five Factor Model (extraversion, consciousness and openness) were implicated in one paper, aligning with Tedeschi and Calhoun’s (1996) findings from the general population. Secondly, SOC was a facilitator in another paper. However, personality is a fluid construct and may represent personality in a snapshot of time influenced by personal circumstances and clinical intervention (Roberts et al., 2017).

### Gender

One study found females with higher PTG than males, which alluded to more women sharing their adverse experiences (Surgenor et al., 2020), implicating social support as a coping strategy. This is consistent with Wu et al.’s (2016) study, showing that women scored higher than men on the ‘relating to others’ subscale of PTG. Social role theory (Eagly, 1997) suggests that men are less likely to seek social support due to stigma around venting of emotions (Chatmon, 2020). Some studies had predominant or only male samples, which was a limitation of this review. Future research should aim to include a representative sample to determine if the relationship between PTG and other variables of interest is reliably mediated by gender. This is important to consider when nearly half of ambulance personnel identify as women (NHS England, 2021).

### Incident characteristics

There was limited evidence from three studies suggesting that incident effect, type or duration were associated with PTG (high or low) and coping style (adaptive or maladaptive). Other studies did not collect demographic information or include this in analysis, and trauma may impact on the quality of memories involving incident effect or duration due to fragmentation (Bedard-Gilligan & Zoellner, 2012), which may limit reliability of findings. Further research in this area would be useful for theory development.

### Methodological limitations and future research

Most studies had excellent methodological quality and utilised the same questionnaire (PTGI) to measure PTG, which enabled the synthesis of findings. However, the sole use of questionnaires is reductionist because complex relationships are reduced to numbers. Data triangulation would provide a more comprehensive understanding of PTG (Noble & Heale, 2019). For example, interviews could be used in conjunction with PTGI scores to enhance the reliability and validity of findings. Some studies had participation bias due to recruitment strategy, such as letter advertisements, or were based on mixed or small samples, reducing the homogeneity and statistical power, potentially impacting findings. Differences in findings could be explained by variance in sample demographics and outcome measures, as studies used various questionnaires to measure coping and resilience. Questionnaires were translated, which enabled comparison of data from a range of study populations. However, it is not clear if this was from an authenticated source. Furthermore, cultural differences highlighted that the ‘paramedic’ was either defined by the individual or a role depending on the study location. For instance, China does not have a ‘paramedic’ occupation; rather this is a collective role made up of nurses, stretcher-bearers and doctors, which may make samples less homogeneous. Future research is needed on PTG using a semi-structured interview or questionnaire with open-ended questions because it is important to explore ambulance personnel’s subjective lived experiences of PTG, rather than generalise findings across diverse study populations. For instance, there is no research exploring incident type and post-traumatic growth in the UK or the intertwined and complex relationship between PTG and PTSD.

## Conclusion

This literature review implicated several facilitators of PTG: adaptive coping, high levels of resilience, personality traits (such as extraversion, openness, conscientiousness, sense of coherence) and shorter emergency incidents are associated with higher levels of PTG in ambulance personnel. PTG ranged from low to moderate (38%–69%). These facilitators could be considered by ambulance services and clinical psychologists to promote PTG when offering support following a trauma. However, findings are mixed and should be interpreted with caution. Further research using a qualitative design or mixed methodology is needed to draw firmer conclusions.

## Author contributions

MA was the lead author and led on the planning of the project, literature search and analysis and synthesis of the findings. AS was the project supervisor and provided feedback on the paper. MA acts as the guarantor for this article.

## Conflict of interest

None declared.

## Ethics

Not required.

## Funding

None.

## Registration and protocol

None.
